# Assembly mechanism of primary forest dominated by habitat filtering in karst degradation region

**DOI:** 10.3389/fpls.2025.1676356

**Published:** 2025-10-29

**Authors:** Wenhui Sun, Lili Yang, Chen Zhang, Gongxiu He, Hu Du, Zhaoxia Zeng, Hao Zhang

**Affiliations:** ^1^ Institute of Subtropical Agriculture, Chinese Academy of Sciences, Changsha, China; ^2^ College of Soil and Water Conservation, Central South University of Forestry and Technology, Changsha, China; ^3^ Huanjiang Agriculture Ecosystem Observation and Research Station of Guangxi, Guangxi Key Laboratory of Karst Ecological Processes and Services, Huanjiang Observation and Research Station for Karst Ecosystems, Chinese Academy of Sciences, Huanjiang, China

**Keywords:** assembly mechanism, average shared variance, plant traits, Phylogenetic signal, Karst region

## Abstract

**Introduction:**

The revelation of the assembly mechanism of plant communities in karst region has crucial implications for the restoration of degraded vegetation. Niche theory and neutral theory are the two main theories to elucidate community assembly of karst plant community. However, the relative significance of habitat filtration and biological action in community assembly remains a topic of debate.

**Methods:**

By using measurement of plant functional traits, detection of phylogenetic signal (K value), and average shared variance, our investigation aimed to ascertain whether species coexistence in community assembly of primary forest is driven by habitat filtering or biotic constraints.

**Results:**

In all 10 plant functional traits, leaf carbon (LC) had the lowest variation coefficient, whereas leaf area (LA) exhibited the highest. Significant phylogenetic signals (*P* < 0.05) were identified for plant LC, LA, wood density (WD), leaf nitrogen (LN) and leaf phosphorus (LP). Phylogenetic signal strength (K < 1) of all traits indicated that the phylogenetic conservation of functional traits is relatively weak and may be influenced by environmental screening or convergent evolution. Both the phylogenetic net relatedness index (NRI) and nearest taxon index (NTI) were negative, indicating a divergent phylogenetic structure. Additionally, with the exception of LA and leaf length-width ratio (L/D), the mean pairwise trait distance indices (SES.PW) were greater than 0, suggesting a tendency towards aggregation in the functional trait structure. Furthermore, average shared variance demonstrated that variation in plant functional trait was predominantly influenced by soil fertility and topography of the sample

**Discussion:**

Our finding indicated that the community assembly of primary forest plant was dominated by habitat filtering, which could significantly promote a more profound comprehension of natural restoration in karst degradation region.

## 1 Introduction

Plant functional traits emerge from prolonged coevolutionary processes between flora and their abiotic matrices, serving as diagnostic indicators for quantifying species’ adaptive strategies under environmental constraints while enabling forecasting of ecosystem responses to anthropogenic perturbations across phytocommunity hierarchies (Violle et al., 2012). The connection between functional traits and environmental gradients serves as a crucial element within the theoretical framework of community assembly (Sundqvist et al., 2013). From an ecological niche perspective, species coexistence or community composition at different scales is not only dependent on environmental factors (climate, soil, topography, and disturbances), but also the effect of trait variation and trait combinations of community species (Lu et al., 2025; Valladares et al., 2015). The interplay between habitat and biotic factors affects the variation of plant functional traits, thereby influencing the processes of environmental sieving and filtering (Tameirão et al., 2021). On one hand, environmental filtering selects species with compatible functional traits for particular conditions, leading to trait similarity among species that coexist in the community (Backhaus et al., 2021). Conversely, the competitive exclusion of species that are too similar within a community decreases the overlap of ecological niches and eases the pressure of resource competition, thus resulting in trait divergence among species in similar habitats (Gewin, 2006). It is therefore proposed that the scope of functional trait values is an outcome of the combined effects of environmental filtering—a process that narrows the range of trait variation among coexisting species—and competition-driven niche differentiation, which serves to broaden the range of trait variation among coexisting species (Carvalho et al., 2006; Gewin, 2006).

Previous researches have indicated that the correlation of traits between species must consider the phylogenetic relationship between species, and test whether the functional traits of species have a phylogenetic signal (Ackerly, 2009). The *K* value method has been widely applied to the correlation analysis of phylogenetic relationships among species due to its simplicity of operation and reliability of the results. Nevertheless, it should be noted that not all functional traits are directly associated with phylogenetic history. Even in the situation where species that are closely related, the heterogeneity of their environments may have an impact that extends far beyond the scope of their evolutionary history if they are located in environments that are markedly different (Sterelny, 2005). Consequently, in some cases, the functional traits of these species exhibit dissimilarities in ecological function or morphology, thus failing to adequately reflect their phylogenetic signals. Moreover, species that are distinct or have evolved differently may display convergent evolution in their morphological structures when they have been adapted to identical environments over an extended period of time (Vidal and Keogh, 2015). Convergent evolution can lead to the emergence of similar functional traits in species that are distantly related (Stern, 2013). However, these traits may not directly reflect their phylogenetic signals. To explore the influence of phylogeny on functional traits, average shared-variance are employed to obtain more accurate relationships between functional traits and phylogeny (Carvalho et al., 2006; Lai et al., 2023).

The phylogeny and functional traits of a community have the potential to shed light on a wide range of ecological processes (Valladares et al., 2008). Webb (2000) blazed a trail by employing phylogenetic trees as a novel tool in the study and examination of community ecology. He subsequently outlined the operational steps for phylogenetic structure analysis, which involves comparing the phylogenetic distances between species with those predicted by the null model. This comparison allows for the determination of whether the distribution pattern of species within the community is characterized by phylogenetic clustering, phylogenetic overdispersion, or randomization. Such an analysis aids in inferring the primary drivers of community assembly. When the development of functional traits is conservative, a comparison with the null model reveals that if community exhibit a phylogenetic clustering distribution pattern, it indicates that in the process of community assembly, similar habitats would select species with closer adaptive ability and kinship to form a community. This observation points to a notable effect of environmental filtering on the system. Conversely, if the community species exhibit a discrete distribution pattern, this indicates that competitive exclusion is the dominant factor (Zhao et al., 2023). Consequently, the community is composed of species with distant affinities. If functional traits are not conserved, a phylogenetic structure that shows dispersion points to environmental filtering as the main driver of community assembly. Conversely, a phylogenetic structure displaying aggregation or randomness indicates that competitive exclusion dominates community assembly (Wilson and Stubbs, 2012). Simultaneously, akin to the structure of species lineages, the distribution pattern of functional traits can also shed light on the role of various ecological processes in community assembly (Wiegand et al., 2017). Specifically, compared to the null model, the overall distribution of functional traits suggests a more pronounced role of environmental filtering. Conversely, a functionally dispersed trait distribution implies that interspecific competition, or similarity limitation, dominates in community assembly (Kunstler et al., 2012).

The karst exposure in southwestern China is approximated to cover an area of 540,000 km², making it one of the most extensive continuous distributions of karstic terrain worldwide (Zhao et al., 2022). The karst ecological environment is typified by pronounced heterogeneity, a significant rate of rock exposure, fragmented and inadequate soil, limited total soil volume and capacity, severe soil erosion, and rock drought (Lu et al., 2023). These inherent environmental stressors foster the evolution of lithophytic plants that exhibit drought resistance, barren resistance, calcium preference, and lithophilic characteristics (Zou et al., 2025). As a result, this leads to the formation of a subtropical karst mixed forest climax community of evergreen and deciduous broad-leaved trees, which is distinct from the non-karst forest climax community at the same latitude (Zou et al., 2023). This community is characterized by a rich composition of tree species, a diverse community structure, and dominant species that are prominently featured (Yi et al., 2024). Although the neutral theory emphasizes the role of random processes in community assembly, in the extremely heterogeneous environment of karst forest, environmental screening may be more explanatory. The integrated analysis of phylogenetic and functional traits can quantify the relative contributions of niche processes and neutral processes, thereby providing an empirical basis for verifying the two theories (Cheng et al., 2025). To date, investigations have been execute on the flora and species composition, shrub phylogenetic structure, and plant physiological traits in this region (Zhang et al., 2025; Zou et al., 2024; Sun et al., 2017). While prior research has employed phylogenetic signals to investigate the evolutionary traits of characteristics in order to elucidate assembly mechanism of community, there has been no integration of phylogeny and functional traits to collectively examine the influence of various ecological processes on the formation of karst forest community (Li et al., 2017). Employing an integration of genealogical-functional trait analysis along with phylogenetically independent comparisons, we aim to address three key scientific inquiries: (1) Whether the phylogenetic conservation of functional traits is sufficient to reveal the environmental screening mechanism of community assembly? (2) How is the kinship structure of functional traits manifested in karst woody plants? (3) What is the comparative significance of phylogenetic and environmental determinants in influencing functional trait variation?

## 2 Materials and methods

### 2.1 Research site

**Figure 1:**
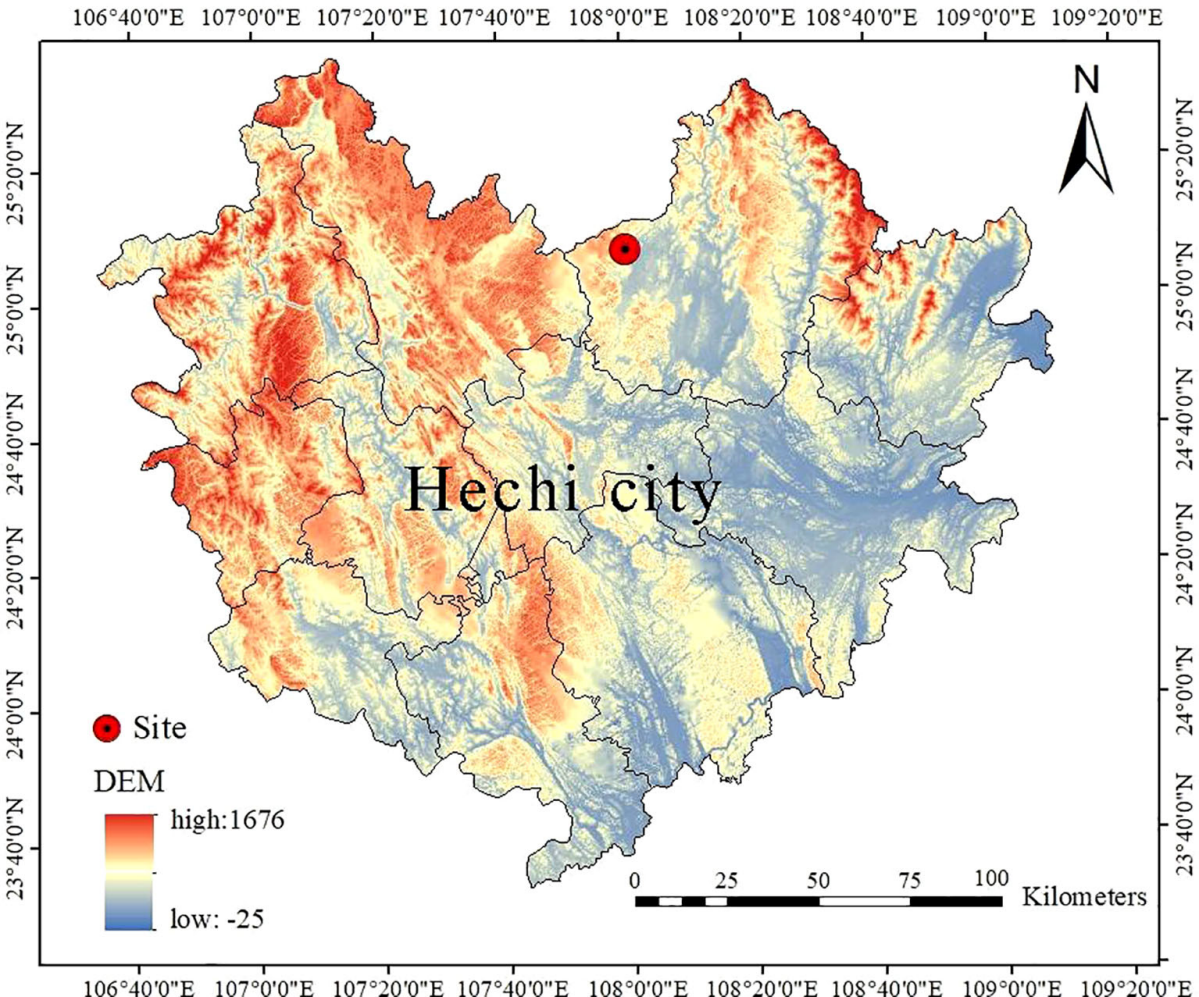
The location of study site.

The research site was located in the Mulun National Nature Reserve (24°44´-25°33´N, 107°51´-108°43´E) within the Hechi City, Guangxi Zhuang Autonomous Region, China (**Figure 1**). The altitude of the research site ranged from 442.6 m to 651.4 m. The region experiences an average annual temperature of 15.7°C, with January and July mean temperatures being 10.1°C and 28.0°C respectively. Annual rainfall is 1,389 mm, and the region receives 4,422 hours of sunshine annually. These characteristics are indicative of a mid-subtropical monsoon climate zone. A total of 6,754 plants with a diameter at breast height (DBH) no less than 1 cm were identified at the research site. The primary species identified within this plot include *Itoa orientalis*, *Bridelia tomentosa*, *Cornus macrophylla*, *Pittosporum kwangsiense* and *Hibiscus sabdariffa*. The geomorphological type of the area is characterized by karstic crested depressions with complex and variable habitat topography (Zhang et al., 2013).

From July to September 2021, we surveyed thirty-five 20 m × 20 m sample plots and each plot was partitioned into sixteen 5 m × 5 m subplots. Herbaceous plants were investigated in full coverage using a 5 m× 5 m small sample plot, with species names, coverage and height recorded to estimate the diversity and biomass of the understory herbaceous layer. However, subsequent functional trait and phylogenetic analyses were only conducted for woody plants (DBH≥1 cm).

### 2.2 Measurement of leaf traits

A sum of 10 plant functional traits was picked for investigation, including leaf area (LA, cm²), leaf thickness (LTH, mm), specific leaf area (SLA, cm^2^·g^-1^), leaf dry matter content (LDMC, g·g^-1^), leaf aspect ratio (L/D), leaf tissue density (LTD, g·cm^-3^), wood density (WD, g·cm^-3^), leaf nitrogen (LN, g·kg^-1^), leaf phosphorus (LP, g·kg^-1^) and leaf carbon (LC, g·kg^-1^). The contents of carbon, nitrogen and phosphorus are vital to the functioning of the biochemical and geochemical cycles (Onoda et al., 2011; Wright et al., 2004; Cornelissen et al., 2003). Following the approaches of Pérez-Harguindeguy et al. (2013), we measured LA, LTH, LDMC, SLA, LTD, LN, LP, and LC.

Based on the species survey data from each plot, we randomly selected 3–5 individuals with a DBH ≥ 1cm. If a plot contained fewer than three individuals, all were chosen. For woody plants present, every individual was sampled. From the outermost edge of each woody plant’s crown, one complete branch was collected from each of the four cardinal directions: east, south, west, and north. These branches were exposed to full sunlight and showed no signs of disease, pest infestation, or epiphytic growth. From each branch, 5–6 leaves were taken, ensuring a total sample size of 20–30 leaves per woody plant. For multi-stem plant individuals, we treat them as an independent sample for processing, ensuring the comprehensiveness and accuracy of data collection.

### 2.3 Measurement of environmental factors

The topographic factor data were obtained through the following methodology: during the construction of the sample plots, a combination of real-time dynamic differencing (RTK) and total station was employed to calculate the average elevation (Alt), slope (Slo), and aspect (Asp) of the sample plots. This was achieved by measuring the elevation of the four points of each 20 m × 20 m sample plot. Additionally, rock outcrop rates (Roc) were estimated through visual inspection within the sample plots.

The soil sampling depth is 0–20 cm of surface soil. For each sample plot, samples are mixed using the five-point method. Eight soil physical and chemical factors were considered, including soil pH (pH), soil organic matter (SOM), soil total nitrogen (STN), soil alkaline nitrogen (SAN), soil total phosphorus (STP), soil effective phosphorus (SAP), soil total potassium (STK), and soil effective potassium (SAK). SOM, pH, STN, SAN, STP, SAP, STK and SAK were determined as described by Xu et al. (2001). In the case of every soil sample, three replications were carried out, and the mean value was taken into account for analysis.

### 2.4 Data analysis

#### 2.4.1 Phylogenetic trees

We conducted a comprehensive investigation into the family, genus, and species of all woody plants within the plots. Utilizing the phylo.maker function from the V.Phylo Maker package (Jin and Qian, 2019), we constructed a phylogenetic tree based on scenario 3 (**Supplementary Figure S1**). Subsequently, branch lengths were assigned in accordance with the BLADJ algorithm to facilitate community phylogenetic analysis. Ten functional traits were chosen for further examination. Using principal component analysis, we identified the principal components that adequately represented all functional traits. The trait matrix was then converted into a distance matrix using the Gowdis distance. This functional trait distance between species served as the measurement standard. Hierarchical clustering analysis was performed on these data, resulting in the generation of a functional trait clustering tree based on the analysis outcomes (**Supplementary Figure S2**) (Yang et al., 2014; Petchey and Gaston, 2002).

#### 2.4.2 Detection of phylogenetic signals

We adopt the *K* value from the Brownian motion evolution model proposed by Blomberg et al. (2003) as an indicator for phylogenetic signal analysis. If *K* > 1, it suggests that functional traits exhibit a stronger phylogenetic signal than what is predicted by the Brownian motion model of evolution. Conversely, *K* < 1, it implies that these traits display a weaker phylogenetic signal compared to the Brownian motion model. *K* = 1 aligns with the predictions of the Brownian motion evolutionary model. *K* approaching 0 suggests the absence of a phylogenetic signal, implying that trait evolution is independent (Liu et al., 2023). The significance of the phylogenetic signals of functional traits can be tested by comparing the actual *K* value of the community with the null model *K* value, which is obtained by randomly substituting the species at the end of the branch of the phylogenetic tree for 999 times. If the actual *K* value is more than the null model *K* value (*P < 0.05*), the phylogenetic signal for the functional trait of the community is considered significant. Otherwise, it is non-significant (Baraloto et al., 2012). The *K* values were calculated using the phylosignal (Keck et al., 2016) function of the R software picante package, as described by Kembel et al. (2010).

#### 2.4.3 Phylogenetic and functional traits structure analysis

The net relatedness index (NRI), net nearest taxa index (NTI) and mean pairwise trait distance (SES.PW) were employed to calculate the phylogenetic and functional trait structure of trees in different quadrats by the R software picante package. Values of NRI and NTI exceeding 0 suggest phylogenetic structure of species aggregation, while those less than 0 indicate phylogenetic structure of species divergence. A value of 0 for both NRI and NTI implies a random distribution of phylogenetic structure of species (Elliott et al., 2016). When SES.PW exceeds 0, it suggests that the functional trait structure is aggregated. Conversely, if SES.PW falls below 0, it implies a divergent functional trait structure. A value of SES.PW equal to 0 indicates a random functional trait structure (Hanz et al., 2022).

#### 2.4.4 Average shared variance analysis

The average shared-variance states that when several predictors explain the same response, their overlapping explanatory power is counted multiple times. After removing this common variance, each predictor’s unique contribution can be gauged to quantify its true importance. With the R function phyloglm.hp (), we can decompose the R² of models fitted by phylolm () or phyloglm (): it returns both individual R² values for environment and phylogeny (summing exactly to the full-model R²) and the shared R² between them, thereby explicitly quantifying the relative roles of evolutionary history and environmental drivers in shaping traits (Lai et al., 2023, 2024).

## 3 Results

### 3.1 Phylogenetic signaling of functional traits

Among the 10 functional traits, LC had the lowest coefficient of variation (4.78%), while LA had the highest (85.34%) (**Table 1**). The *K* values of all functional traits exhibited a range from 0.162 (SLA) to 0.505 (LN), all of which were less than 1 (**Table 2**). A comparison of the actual values with the null model revealed the presence of significant phylogenetic signals (*P* < 0.05) for five functional traits (LA, WD, LN, LP and LC). However, the *K* values were found to be relatively low. In contrast, no significant phylogenetic signals (*P* > 0.05) were identified for the remaining five functional traits (L/D, LTH, LDMC, LTD and SLA).

**Table 1:** Descriptive statistics of plant functional traits measured from sampled 109 species.

Trait	Unit	Mean ± SE	Max	Min	CV
L/D	—	2.475 ± 0.050	4.275	1.095	20.97
LA	cm^2^	44.759 ± 36.590	251.122	1.857	85.34
LTH	mm	0.253 ± 0.009	0.547	0.08	35.52
WD	g·cm^-3^	0.491 ± 0.011	0.756	0.105	24.20
SLA	cm^2^·g^-1^	169.748 ± 50.960	393.638	72.528	31.34
LDMC	g·g^-1^	0.380 ± 0.060	0.651	0.249	16.16
LTD	g·cm^-3^	0.310 ± 0.012	0.932	0.122	40.31
LN	g·kg^-1^	22.16 ± 5.600	36.03	10.4	26.46
LP	g·kg^-1^	1.13 ± 0.040	3.0	0.42	36.42

L/D, Leaf length-width ratio; LA,Leaf area; LTH, Leaf thickness; WD, Wood density; SLA, Specific leaf area; LDMC, Leaf dry matter content; LTD, Leaf thickness density; LN, Leaf nitrogen; LP, Leaf phosphorus; LC, Leaf carbon.

**Table 2:** The phylogenetic signal of functional traits in karst plant community.

Functional traits	*K* value	*P* value
Leaf length-width ratio	0.240	0.157
Leaf area (cm^2^)	0.258	0.048
Leaf thickness (mm)	0.236	0.065
Wood density (g·cm^-3^)	0.349	0.005
Specific leaf area (cm^2^·g^-1^)	0.162	0.783
Leaf dry matter content (g·g^-1^)	0.165	0.675
Leaf tissue density (g·cm^-3^)	0.228	0.128
Leaf nitrogen content (mg·g^-1^)	0.505	0.001
Leaf phosphorus content (mg·g^-1^)	0.291	0.020
Leaf carbon content (mg·g^-1^)	0.429	0.001

The phylogenetic signals of leaf traits are considered statistically significant when the *P*-value is ≤ 0.05.

### 3.2 Phylogenetic and functional trait structure

**Figure 2:**
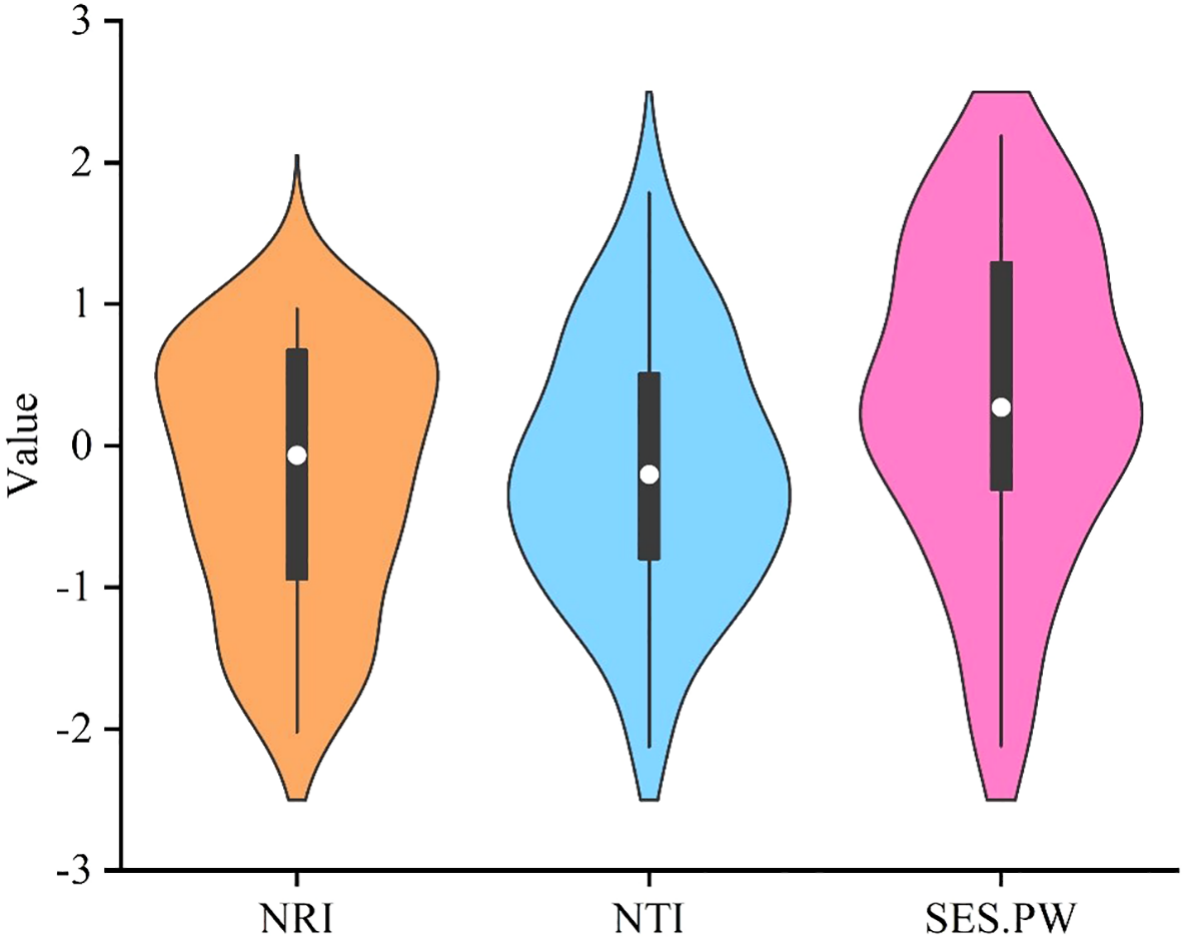
Phylogenetic community structure index and functional trait community structure index of karst plant community.

**Figure 3:**
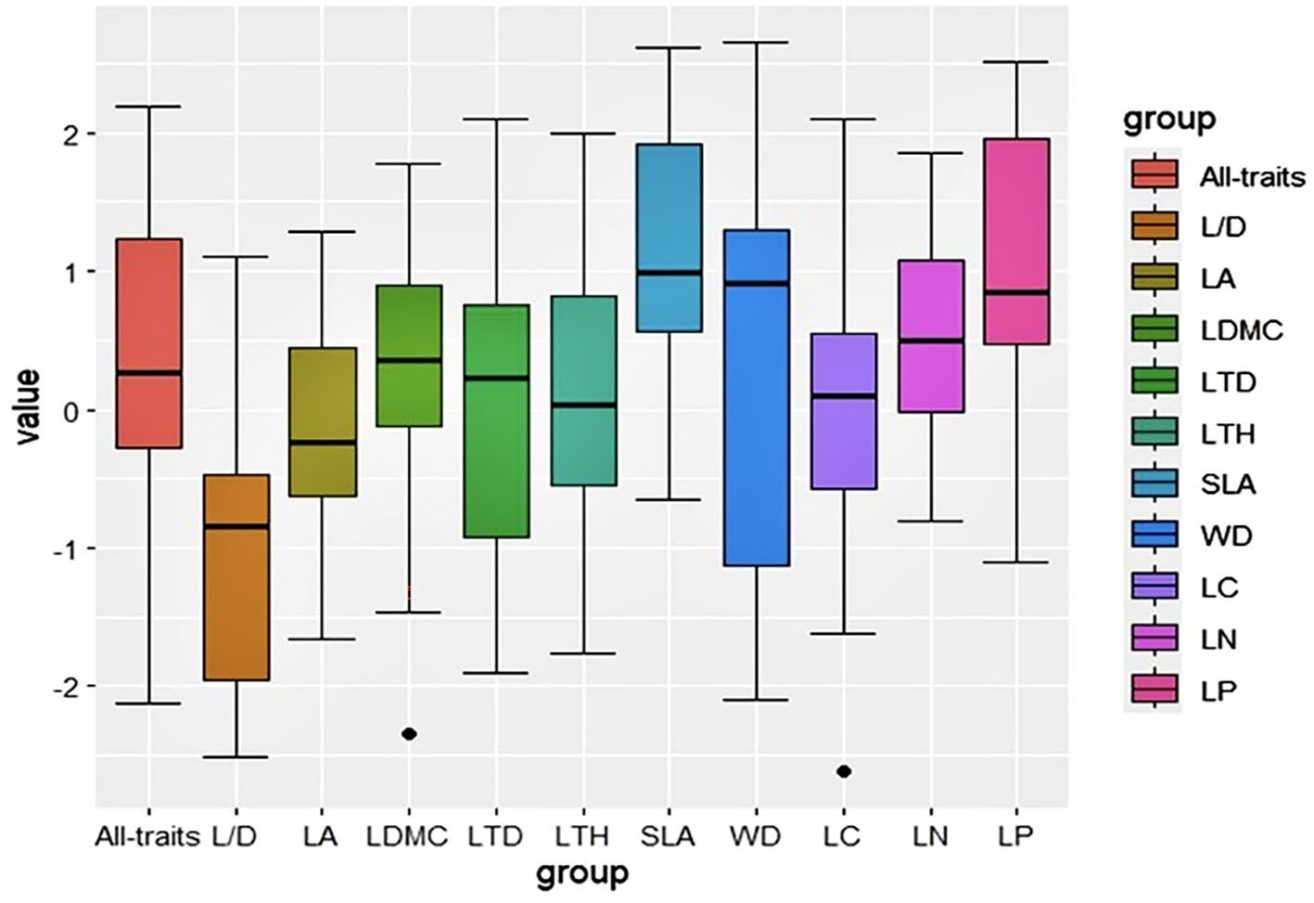
Functional trait community structure index of different traits in karst plant community.

The phylogenetic structure indices, namely NRI and NTI, for the 35 plots reveal a mean value less than zero (**Figure 2**). The mean pairwise trait distance index (SES.PW) within the functional trait structure index indicates that, with the exception of LA and L/D, most plant functional traits exhibit SES.PW larger than zero, thereby signifying clustering in functional trait structures (**Figure 3**).

### 3.3 Individual explanatory rates of phylogenetic development, topographic and soil factors for functional traits

**Figure 4:**
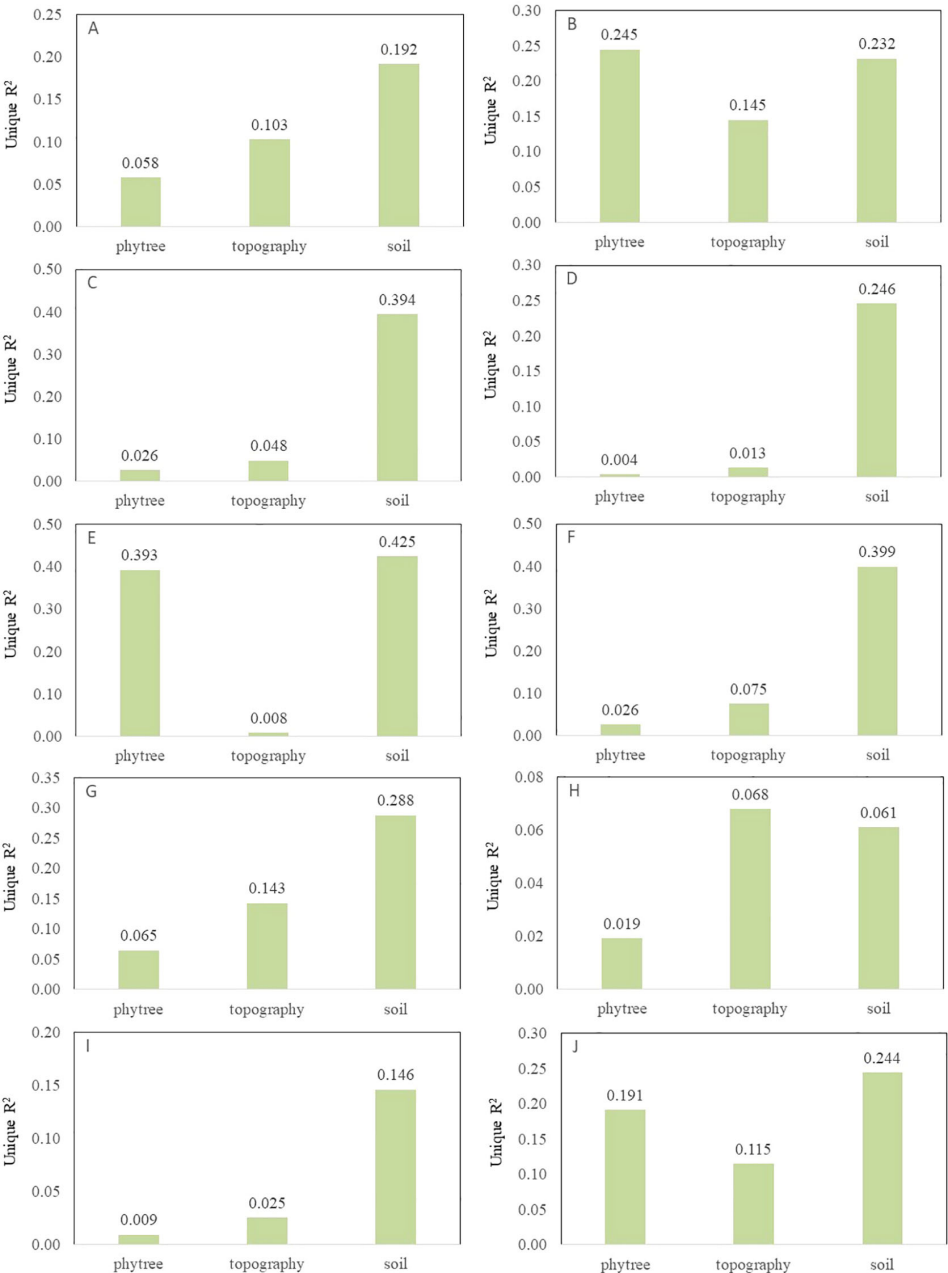
The individual explanatory rates of phylogenetic development, topographic and soil factors for 10 plant functional traits (**(A)** leaf area, **(B)** leaf carbon, **(C)** leaf length-width ratio, **(D)** leaf dry matter content, **(E)** leaf nitrogen, **(F)** leaf phosphorus, **(G)** leaf tissue density, **(H)** leaf thickness, **(I)** specific leaf area, **(J)** wood density).

Overall, environmental factors (topography and soil) have a greater impact on all functional traits than phylogeny (**Figure 4**). Among them, phylogeny contributes significantly to the variations of LC, LN and WD, while environmental factors have a greater impact on LA, L/D, LDMC, LP, LTD and SLA. SOM, STN, STP and SAP are the main soil factors influencing plant functional traits, especially having a relatively high explanatory power for nutrient-related traits such as LN and LP (**Supplementary Figure S3**). Topographic factors such as Asp, Slo and Roc also make certain contributions to some traits (such as LTD and WD), but the overall contribution rate is lower than that of soil factors.

## 4 Discussion

### 4.1 Phylogenetic conservatism and convergence of functional traits

Previous studies have shown that the phylogenetic structure and functional trait structure behave consistently when the functional traits exhibit characteristics related to its evolutionary history, which is an important prerequisite for using phylogenetic structure to explore the mechanism of community assembly (Satdichanh et al., 2015; Garnier and Navas, 2012). Significant phylogenetic signals (P<0.05) combined with K values (K<1) indicate that despite phylogenetic conservation, environmental screening may have weakened the direct effect of kinship, leading to trait convergence. Our results align with the findings of Yunzhi et al. (2018) in a subtropical forest community in the Badagong Mountains of Hunan. It suggests that functional traits are evolutionarily conserved and that the community phylogenetic pattern should be consistent with the functional trait pattern. Nevertheless, the study of community assembly in fragile habitats, such as deserts and sand dunes, has demonstrated that not all functional traits of plants exhibit significant developmental signals (Li and Sun, 2017; Li et al., 2014). The phylogenetic signals for other functional traits in our study were not statistically significant, developmental conservatism was weak, and plant functional traits exhibited convergence on evolutionary pathways, suggesting that environmental factors may exert a considerable influence on the observed variation in these functional traits. This may also be attributed to the inherent difficulty in detecting significant phylogenetic signals for certain plant traits, as well as the concurrent impact of evolutionary genetics and environmental factors on plant traits (Swenson, 2013; Remington and Purugganan, 2003). The *K* values of the 10 chosen functional traits were all below 1, which indicates that the phylogenetic signals of the functional traits of the species in the sample plots were less pronounced than anticipated by the Brownian motion model of trait evolution, suggesting a weaker developmental conservatism and a convergence of the ecological traits of the plant communities. This might be due to the more specific habitat attributes of the karst region and the greater environmental heterogeneity, which require plants to adapt to harsh conditions. The functional traits are therefore subject to adaptation in order to suit the specific characteristics of the habitat (Akram et al., 2023; Kembel and Cahill, 2011). For a better grasp of the processes involved in community assembly, the phylogenetic and functional trait patterns of the community need to be analyzed together.

### 4.2 The impact of environmental factors on functional traits

The findings of previous studies have demonstrated that there is no straightforward one-to-one correspondence in terms of the relationship of functional traits and relatedness (Wang et al., 2022). The integration of these two factors allows for a more thorough comprehension of the elements underlying community assembly. This can be achieved by examining the phylogenetic signals of functional traits, analyzing their evolutionary characteristics, and investigating the coupling between functional traits within the context of the phylogeny in question (Díaz et al., 2013). It has been highlighted that the influence of phylogenetic relationships on functional traits can be negated through the utilization of phylogenetically independent comparison methodologies (Burns and Strauss, 2012). In this study, following the removal of phylogenetic factors through phylogenetically independent comparison of LA, WD, LN, LP, and LC, it was observed that the coupling effect between functional traits was diminished. This outcome provides extra comprehension regarding the function of phylogeny in the extended evolution of functional traits, matching the conclusions reached by Kraft and Ackerly (2010).

Average shared-variance analysis revealed that phylogeny is a significant factor influencing the variation of functional traits in plants. Furthermore, environmental factors (topography and soil) have a greater impact on all functional traits than phylogeny. It has been demonstrated that, at the macro scale, environmental factors, such as climatic conditions, exert a predominant influence on the dispersion of plant functional traits (Zimmermann et al., 2010). Conversely, at the micro scale, topographic factors, including soil nutrients and slope, generally dictate the distribution of plant functional traits (Shipley et al., 2006). Previous studies have demonstrated that alterations in slope have a pronounced impact on soil water content and the distribution of nutrients (e.g., carbon, nitrogen, phosphorus) (Li et al., 2021). Additionally, bare karst surfaces have been shown to result in significant nutrient loss, which in turn affects plant functional traits (Green et al., 2019). The results of our study concur with these findings. The assembly of community can be elucidated by examining the interplay between phylogeny and plant functional traits, as this reveals the mechanisms underlying species coexistence and maintain biodiversity (Cadotte et al., 2009; Long et al., 2025). We focused on functional traits, and future research could expand the scope to encompass a more diverse array of functional traits and an even greater spatial scale.

### 4.3 Revealing the assemble mechanism of karst forests from the perspective of function-phylogenetic development

Plant functional traits are crucial for distinguishing the ecological niche of species (Funk et al., 2017). The process of phylogeny has a major influence on the evolution of functional traits (Martiny et al., 2015). The joint phylogenetic and functional trait structures can provide a more comprehensive understanding of community information, thus making it essential to combine the two in the study (Xu et al., 2017). In this study, although the overall NTI/NRI is less than 0, many values are close to 0 and the difference from the null model is not significant. Describing it solely by “divergence” is not accurate enough. Combining the results of SES.PW, we are more inclined to believe that the phylogenetic structure of the karst plant community as a whole deviates from the random distribution, but the degree of this deviation is relatively weak. This might reflect the combined effect of environmental filtering and competitive exclusion: environmental filtering screens out species with similar functional characteristics, while competitive exclusion leads to the differentiation of coexisting species in certain features (Yang et al., 2014; Liu et al., 2019). At the same time, we also recognize that future research can further expand the sample size or incorporate more ecological process analyses to more clearly reveal the assembly mechanism of karst plant communities.

This study focuses on the assembly mechanisms of karst forest communities and delves into three core scientific questions. Firstly, it was found that although functional traits exhibit a certain degree of phylogenetic conservatism, the signal strength is generally low, indicating that environmental filtering plays a more crucial role in community assembly. For instance, the coefficient of variation of leaf carbon content (LC) is the lowest (4.78%), suggesting that plants adapt to drought and stress environments through high carbon content. Secondly, the phylogenetic structure of karst forest communities shows significant species divergence (both NRI and NTI are negative), while the functional trait structure is mainly aggregated (most SES.PW values are greater than 0). This inconsistency may result from the combined effects of environmental filtering and competitive exclusion: despite the distant phylogenetic relationships among plants, their functional traits tend to converge, reflecting that plants’ adaptation to karst environments is more driven by environmental pressure than genetic constraints. Finally, environmental factors have higher explanatory power for the variation in functional traits than phylogenetic factors. However, phylogenetic history still has some influence on certain functional traits (such as leaf nitrogen and carbon), indicating that the evolutionary history of plants still has a fundamental shaping effect on their functional traits.

This study reveals that the assembly of karst forest communities is mainly driven by environmental filtering, providing theoretical support for the restoration of degraded karst vegetation and emphasizing the priority of selecting plants with functional traits adapted to karst environments. At the same time, it highlights the importance of integrating phylogenetic and functional trait information in the study of plant community assembly. Future research could be expanded to larger spatial scales, combined with more functional traits and experimental ecological methods, to further explore the complex mechanisms of karst forest community assembly and provide more operational guidance for ecological restoration practices.

## Data Availability

The raw data supporting the conclusions of this article will be made available by the authors, without undue reservation.
