# Supplementary material for: Assembly mechanism of primary forest dominated by habitat filtering in karst degradation region
Source: Front Plant Sci. 2025 Oct 29;16:1676356. doi: 10.3389/fpls.2025.1676356 (PMC12605185; doi:10.3389/fpls.2025.1676356)
Supplement: Supplementary file 1 [file DataSheet1.docx]

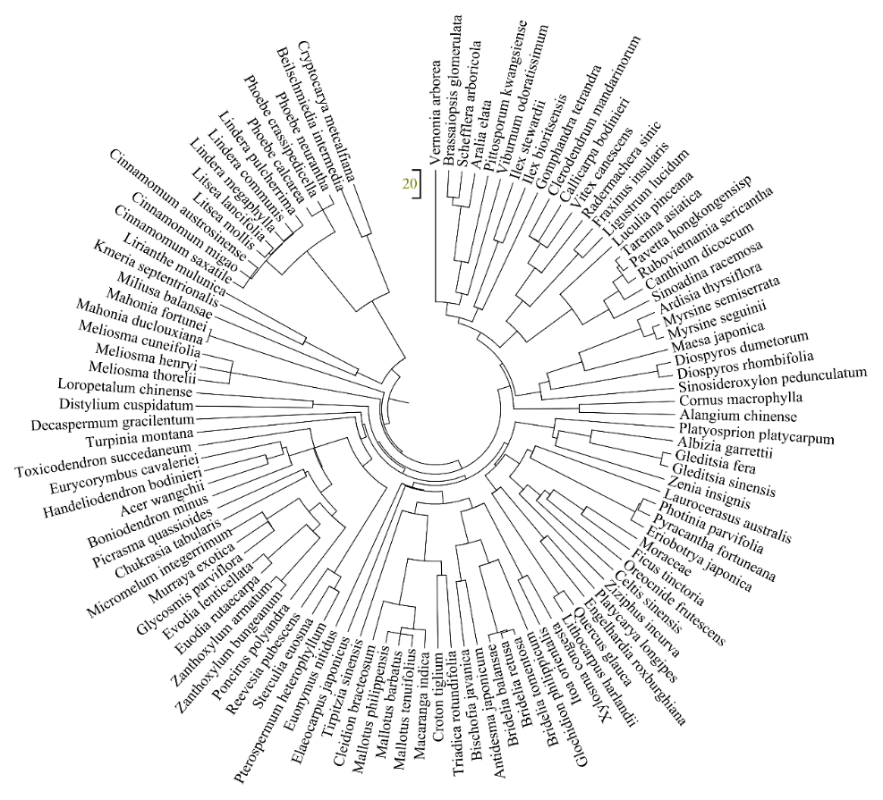


**Figure S1** Phylogeny tree of karst plant community


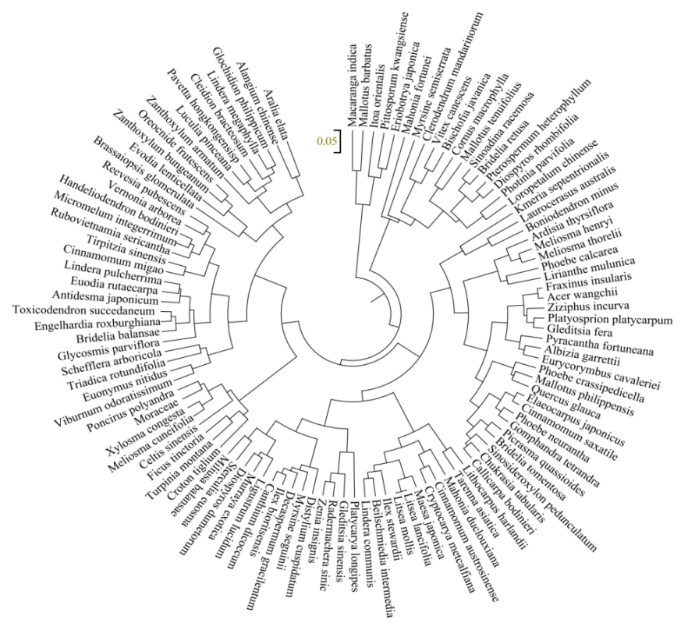


**Figure S2** The clustering functional traits tree of karst plant community


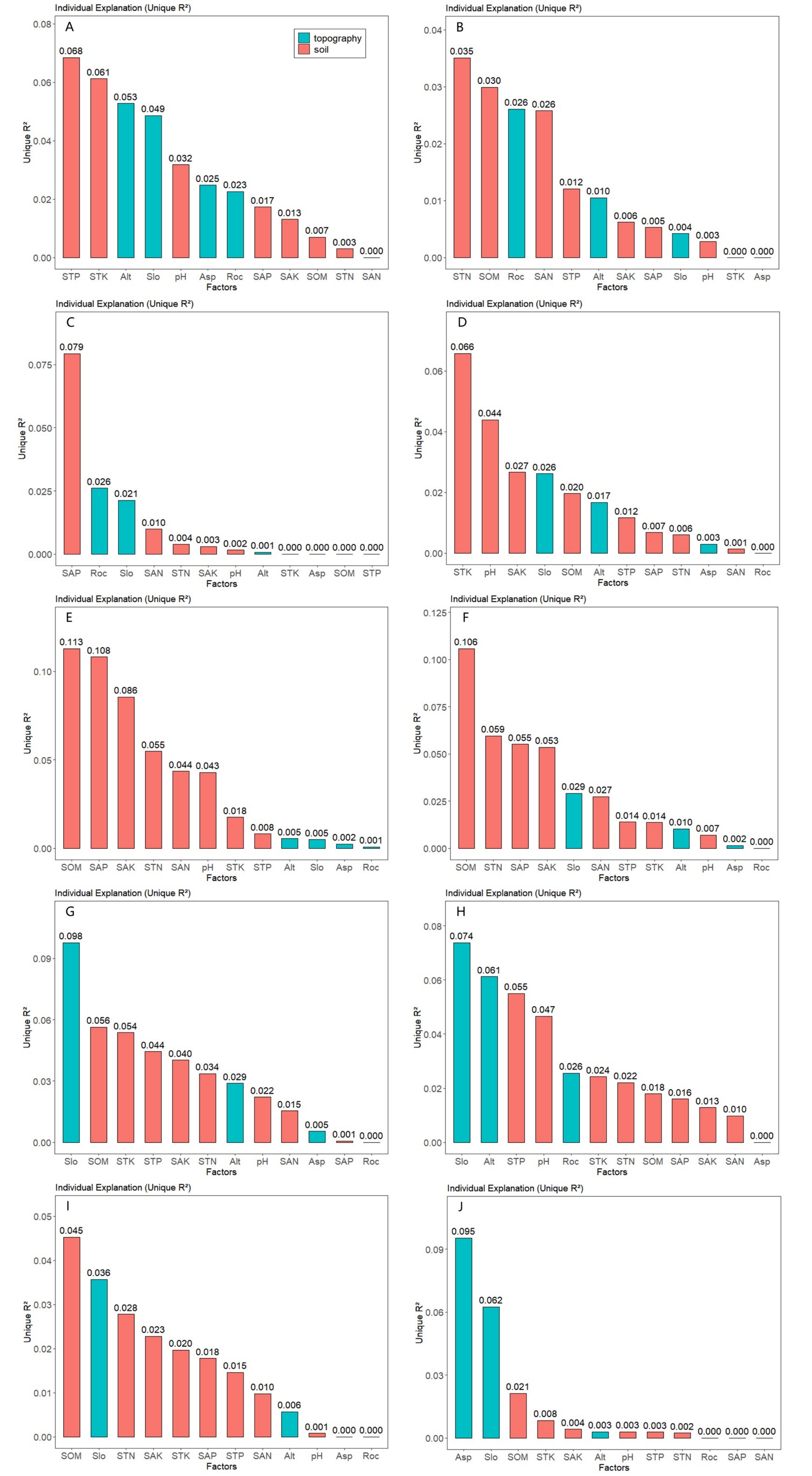


**Figure S3** The individual explanatory rates of topographic and soil factors for 10 plant functional traits (A leaf area, B leaf carbon, C leaf length-width ratio, D leaf dry matter content, E leaf nitrogen, F leaf phosphorus, G leaf tissue density, H leaf thickness, I specific leaf area, J wood density)
